# Development of Neuroblastoma During Growth Hormone Therapy for Short Stature in a Girl With Mosaic Turner Syndrome

**DOI:** 10.7759/cureus.77747

**Published:** 2025-01-20

**Authors:** Takaya Honda, Katsuyuki Tanaka, Taiki Honma, Takuya Kamio, Masaharu Akiyama

**Affiliations:** 1 Pediatrics, The Jikei University School of Medicine, Tokyo, JPN

**Keywords:** anaplastic lymphoma kinase gene, growth hormone therapy, imaging study, mosaic turner syndrome, neuroblastoma

## Abstract

We report the case of a girl with mosaic Turner syndrome who developed abdominal neuroblastoma during growth hormone (GH) therapy for short stature. The patient underwent chemotherapy for the unresectable tumor but died at the age of 10 years due to multiple metastases, five years and 10 months after the diagnosis of neuroblastoma. Although the risk of neuroblastoma does not increase in patients with Turner syndrome, the need for pre-treatment imaging studies for tumor screening remains controversial. However, our experience with this patient highlights the potential value of performing imaging studies both before and during GH treatment.

## Introduction

Growth hormone (GH) therapy is commonly used to enhance the height potential of patients with Turner syndrome [1]. During this treatment, GH is converted to somatomedin C (insulin growth factor 1) in the liver, which promotes chondrocyte proliferation and elongation of the long bones. However, GH therapy is contraindicated in patients with malignant tumors due to its proliferative effects. This case report describes a girl with mosaic Turner syndrome who developed neuroblastoma one year after the initiation of GH therapy.

## Case presentation

A four-year-old girl with mosaic Turner syndrome presented to Jikei Katsushika Hospital in Tokyo, Japan, where she had been undergoing GH therapy for one year to improve her height. She complained of abdominal pain and nausea. A palpable mass was detected in the upper abdomen, and ultrasonographic examination revealed an 8 cm mass. Consequently, the patient was referred to Jikei University Hospital in Tokyo for further evaluation. The patient’s medical history was significant. Cytogenetic analysis, performed at the age of one year and 10 months due to short stature (−3.34 SD), identified abnormal karyotypes, including 45,X 8/30 cells and 47,XXX 22/30 cells, confirming a diagnosis of mosaic Turner syndrome. She showed no evidence of a pterygoid neck or cubitus valgus, features commonly associated with Turner syndrome. Additionally, abdominal ultrasonography performed before the initiation of GH therapy demonstrated no renal morphological abnormalities such as horseshoe kidneys or no adrenal morphological abnormalities typically linked to the condition.

**Figure 1:**
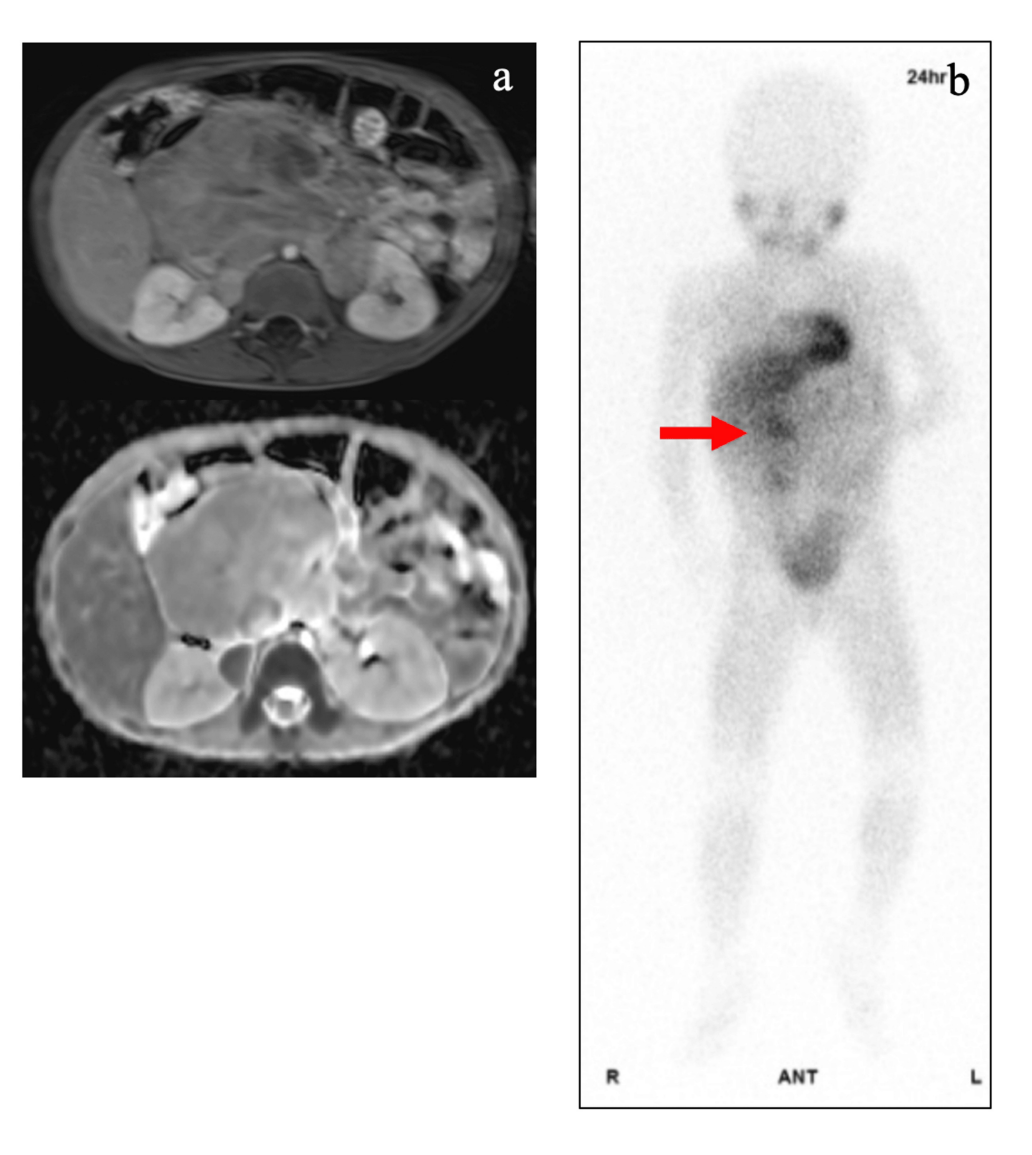
Radiological findings of the abdominal tumor Magnetic resonance imaging of the abdomen (a) shows a large tumor involving the aorta, intra-abdominal artery, and bilateral renal arteries. T1-weighted volumetric interpolated breath-hold examination and apparent diffusion coefficient images are shown in the upper and lower columns, respectively. 123I-metaiodobenzylguanidine scintigraphy (b) shows the absence of hotspots, except for the primary lesion. The red arrow indicates the accumulation of tumor lesions.

The patient was admitted to the hospital and underwent laboratory examinations. Results showed elevated levels of tumor markers, including serum neuron-specific enolase (NSE) (55.0 ng/mL), urinary vanillylmandelic acid (15.5 μg/mg/creatinine), and urinary homovanillic acid (48.3 μg/mg/creatinine). Magnetic resonance imaging of the abdomen revealed a massive tumor involving the aorta, celiac artery, and the right and left renal arteries (Figure 1a). Scintigraphic examination showed the accumulation of 123I-metaiodobenzylguanidine in the mass (Figure 1b). 

**Figure 2:**
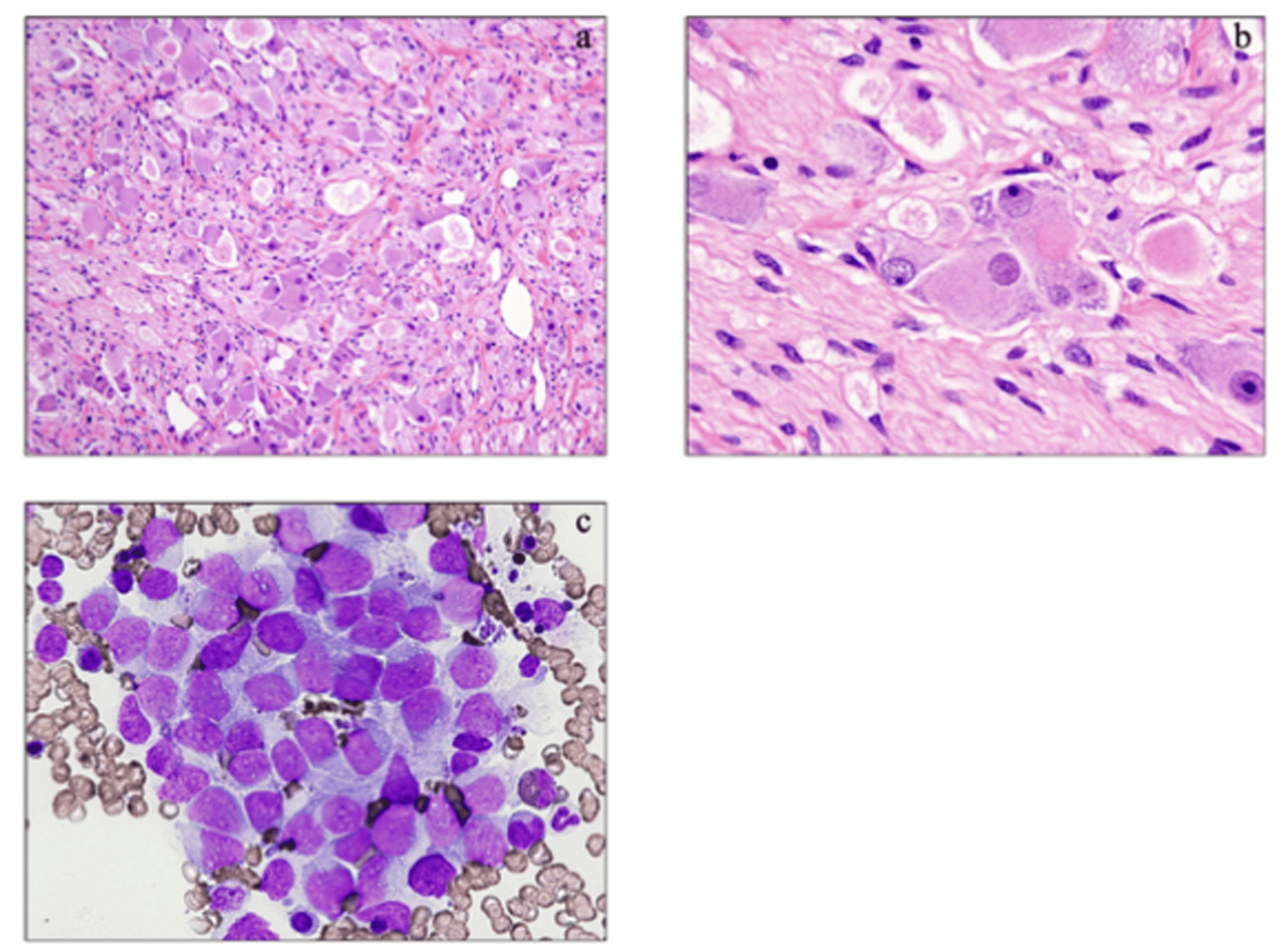
Pathological findings of the tumor The microscopic appearance of the tumor obtained at biopsy (hematoxylin-eosin staining when the patient was aged four years (original magnification (a) ×100 and (b) ×400) is consistent with the characteristics of a ganglioneuroma. (c) The microscopic appearance of bone marrow metastases was obtained by bone marrow aspiration (May-Giemsa staining; original magnification ×400).

A tumor biopsy revealed a histologically complex proliferation of Schwann cell bundles with spindle-shaped nuclei and a small population of differentiated ganglion cells (Figures 2a, 2b). These findings were consistent with the characteristics of ganglioneuroma. The proliferative cells were immunologically positive for S-100 protein, neurofibromin, and NSE-g, whereas nuclear mitosis was not observed (0/10 high-power field). Cytogenetic analysis revealed the following karyotypes: 45,X (8/30) and 47,XXX (22/30) in blood cells and 45,X (17/20) and 47,XXX (3/20) in tumor cells. Moreover, the tumor cells exhibited DNA diploidy but no MYCN amplification. The patient was classified as having intermediate-risk neuroblastoma, according to the International Neuroblastoma Risk Group Staging System [2]. This classification was based on the following factors: localized tumor without distant metastasis (stage L2), the presence of image-defined risk factors, age of >18 months, the presence of undifferentiated neuroblastoma cells, and the absence of MYCN amplification.

The patient initially underwent chemotherapy using the Children’s Oncology Group (COG) A3961 regimen [3]. As the tumor was unresectable but considered stable, 13-cis-retinoic acid was continued as maintenance therapy after eight cycles of the COG A3961 regimen. However, the patient showed signs of disease progression at six years and eight months. This was indicated by elevated levels of serum NSE, urinary vanillylmandelic acid, and homovanillic acid. Contrast-enhanced magnetic resonance imaging and 123I-metaiodobenzylguanidine scintigraphy detected an enlarged abdominal neuroblastoma and metastases to the bone marrow (Figure 2c) and spine. To treat the tumor, several chemotherapeutic regimens were initiated, including 26 cycles of the topotecan and cyclophosphamide regimen [4], at the age of seven years and four months. However, at nine years, the patient was found to be refractory to chemotherapy. Hence, a comprehensive genetic analysis of tumor cells was performed using the FoundationOne® Liquid CDx assay to determine new effective therapeutic agents. This analysis identified the R1275Q variant of the anaplastic lymphoma kinase gene (ALK) (variant allele frequency: 33.0%). However, the patient died of progressive disease at the age of 10 years.

## Discussion

The patient was a girl with mosaic Turner syndrome who developed an abdominal neuroblastoma during the course of GH therapy. Her chromosomal karyotypes included a mix of 45,X and 47,XXX, a condition known as mosaicism with triple X [1]. This karyotype is observed in approximately 3% of patients with Turner syndrome and is typically associated with a mild phenotype [1]. Regarding the association between X chromosome abnormalities and neuroblastoma, no cases of neuroblastoma have been reported in patients with either triple X syndrome or Klinefelter syndrome [5]. However, including the present case, 19 individuals with Turner syndrome complicated by neuroblastoma have been reported (Table 1) [6-20]. These findings suggest that X chromosome aneuploidy may play a role in neuroblastoma development. Compared with neuroblastoma in patients without an underlying condition, neuroblastoma associated with Turner syndrome is typically diagnosed at an older age, has a favorable prognosis due to the presence of differentiated neuroblastoma, and carries a low risk of poor outcomes, often owing to the potential for radical tumor resection. The poor outcome in the present patient was attributed to several factors: the tumor’s involvement with major blood vessels and nerves, which made surgical removal challenging; the presence of undifferentiated neuroblastoma within the heterogeneous tumor; and the residual high-grade neuroblastoma with an ALK mutation (R1275Q) that was refractory to chemotherapy and led to multiple metastases. Due to limited biopsy tissue available and the patient’s subsequent death while under the best supportive care at another hospital, it remains unclear whether the ALK gene R1275Q mutation was present at diagnosis or if it emerged as a new clone during treatment.

**Table 1:** Reported cases of neuroblastoma in patients with Turner syndrome GH: growth hormone; mo: months; y: years; wk: weeks; F: female

Patient	Age	Sex	Karyotype	Diagnosis	Primary lesion	Additional information	References
1	4 mo	F	45,X	Ganglioneuroma	Adrenal		Miller et al. (1968) [6]
2	4 y	F	45,X	Ganglioneuroma	Adrenal		Wertelecki et al. (1970) [7]
3	4 y	F	45,X	Sympathoblastoma stage A	Retroperitoneum		Maximilian et al. (1983) [8]
4	1 y	F	45,X	Neuroblastoma	Adrenal	Opsomyoclonus	Warrier et al. (1984) [9]
5	1 y	F	45,X	Neuroblastoma	Adrenal		Fisher et al. (1994) [10]
6	10 y	F	45,X	Ganglioneuroma	Adrenal	Diagnosed during GH	Matsuoka et al. (1997) [11]
7	1 wk	F	45,X	Neuroblastoma	Adrenal		Blatt et al. (1997) [12]
8	4 y	F	46,X,i(X)(q10)	Ganglioneuroma	Adrenal		Blatt et al. (1997) [12]
9	4 y	F	46,X/45,X	Neuroblastoma	Thoracic		Blatt et al. (1997) [12]
10	16 y	F	45,X	Neurofibrosarcoma with ganglioneuroma	Retroperitoneum/adrenal		Blatt et al. (1997) [12]
11	1 y	F	45,X	Ganglioneuroblastoma	Adrenal	Li-Fraumeni syndrome	Pivnick et al. (1998) [13]
12	6 y	F	45,X	Ganglioneuroma	Adrenal		Sasaki et al. (2000) [14]
13	31 y	F	Turner phenotype	Ganglioneuroma	Adrenal	Screening before GH	Stasik et al. (2005) [15]
14	15 y	F	Turner phenotype	Ganglioneuroma	Adrenal		Kamoun et al. (2010) [16]
15	4 y	F	45,X	Ganglioneuroblastoma	Presacral	Screening before GH	Pinsker et al. (2012) [17]
16	11 y	F	45,X	Ganglioneuroma	Adrenal	Diagnosed during GH	Wikiera et al. (2013) [18]
17	1 y	F	Turner phenotype	Neuroblastoma	Adrenal		Jessop et al. (2022) [19]
18	11 y	F	45,X/46,X,+mar	Ganglion neuroblastoma	Adrenal		Yang et al. (2022) [20]
19	4 y	F	45,X/47,XXX	Ganglioneuroblastoma	Adrenal	Diagnosed during GH	present article

A cohort study in the United Kingdom involving 3,425 women with Turner syndrome showed an increased risk of developing meningioma, brain tumor, bladder cancer, melanoma, and corpus uteri cancer but did not identify an increased risk for neuroblastoma [21]. With regard to the relationship between GH therapy and malignancy, an international cohort study revealed that GH therapy does not significantly increase the risk of malignancy in patients without pre-existing risk factors [21]. However, the risk was significantly elevated in patients with RASopathies, such as Noonan syndrome, or with DNA repair deficiencies, such as Fanconi anemia and Bloom syndrome [22]. Moreover, a cohort study of 5,220 patients with Turner syndrome undergoing GH therapy observed the development of malignancies, but neuroblastoma did not develop in 10 patients who developed malignancies [23]. The researchers concluded that GH therapy does not significantly increase the overall rate of malignancy. However, patients undergoing GH therapy demonstrated a higher rate of malignancy (standard incidence ratio, 2.1) compared with healthy controls. Further study is needed to determine whether GH therapy for patients with Turner syndrome may increase the risk of malignancy.

Several cases of GH therapy-related neuroblastoma have been reported in patients with Turner syndrome [11,15,17,18]. One case involved a 10-year-old girl who developed a primary left adrenal ganglioneuroma after undergoing GH therapy for 3.5 years [11]. Another patient was a 31-year-old woman who developed a ganglioneuroma after completing a 16-year GH therapy [15]. In a four-year-old girl, a ganglioneuroma of the primary left adrenal gland developed, prompting her physicians to recommend screening for neuroblastoma after the diagnosis of Turner syndrome and prior to the initiation of GH therapy [17]. Moreover, a six-year-old girl with Turner syndrome was found to have a ganglioneuroma through an abdominal ultrasonographic examination conducted before GH therapy; this case report suggested that abdominal ultrasonographic examination should be performed after the diagnosis of Turner syndrome and before GH therapy to accurately evaluate the association of Turner syndrome with neurogenic tumors [14]. However, routine screening for neuroblastoma remains a topic of debate due to the rarity of both neuroblastoma and ganglioneuroma, the latter often being a biochemically inactive form of neuroblastoma. Additionally, existing data suggest that the incidence of neuroblastoma does not increase during the course of GH therapy [17]. Based on our experience with the present patient, we recommend that appropriate imaging studies should be considered both before and during GH treatment to monitor and manage the short stature of patients with Turner syndrome.

## Conclusions

We have reported the case of a girl with mosaic Turner syndrome in whom neuroblastoma developed one year after the start of GH therapy for short stature. Nineteen cases, including the present case of Turner syndrome complicated by neuroblastoma, have been reported. However, there is no definitive risk of developing neuroblastoma in patients with Turner syndrome, and the need for imaging studies at the time when Turner syndrome is diagnosed and before GH treatment begins is controversial. Based on our experience with the present case, we recommend that appropriate imaging studies should be considered both before and during GH treatment to monitor and manage the short stature of patients with Turner syndrome.

## References

[1] Gravholt CH, Andersen NH, Conway GS (2017). Clinical practice guidelines for the care of girls and women with Turner syndrome: proceedings from the 2016 Cincinnati International Turner Syndrome Meeting. Eur J Endocrinol.

[2] Monclair T, Brodeur GM, Ambros PF (2009). The International Neuroblastoma Risk Group (INRG) staging system: an INRG Task Force report. J Clin Oncol.

[3] Baker DL, Schmidt ML, Cohn SL (2010). Outcome after reduced chemotherapy for intermediate-risk neuroblastoma. N Engl J Med.

[4] Ashraf K, Shaikh F, Gibson P, Baruchel S, Irwin MS (2013). Treatment with topotecan plus cyclophosphamide in children with first relapse of neuroblastoma. Pediatr Blood Cancer.

[5] Satgé D, Moore SW, Stiller CA (2003). Abnormal constitutional karyotypes in patients with neuroblastoma: a report of four new cases and review of 47 others in the literature. Cancer Genet Cytogenet.

[6] Miller RW, Fraumeni JF Jr, Hill JA (1968). Neuroblastoma: epidemiologic approach to its origin. Am J Dis Child.

[7] Wertelecki W, Fraumeni JF Jr, Mulvihill JJ (1970). Nongonadal neoplasia in Turner's syndrome. Cancer.

[8] Maximilian C, Dumitriu L, Garoiu M (1983). Turner's syndrome with sympathoblastoma. Endocrinologie.

[9] Warrier RP, Kini R, Besser A, Wiatrak B, Raju U (1985). Opsomyoclonus and neuroblastoma. Clin Pediatr (Phila).

[10] Fisher PG, Wechsler DS, Singer HS (1994). Anti-Hu antibody in a neuroblastoma-associated paraneoplastic syndrome. Pediatr Neurol.

[11] Matsuoka H, Shibata E, Ikezaki A, Kim HS, Yamazaki K, Murata M (1997). Ganglioneuroma of left adrenal gland in a patient with Turner syndrome during growth hormone therapy. Acta Paediatr Jpn.

[12] Blatt J, Olshan AF, Lee PA, Ross JL (1997). Neuroblastoma and related tumors in Turner's syndrome. J Pediatr.

[13] Pivnick EK, Furman WL, Velagaleti GV, Jenkins JJ, Chase NA, Ribeiro RC (1998). Simultaneous adrenocortical carcinoma and ganglioneuroblastoma in a child with Turner syndrome and germline p53 mutation. J Med Genet.

[14] Sasaki Y, Nakayama H, Ikeda M (2000). Turner syndrome and ganglioneuroma. J Pediatr Hematol Oncol.

[15] Stasik CN, Giordano TJ, Gauger PG (2005). Ganglioneuroma manifesting as an incidental adrenal mass in an adult with Turner's syndrome. Endocr Pract.

[16] Kamoun M, Mnif MF, Rekik N (2010). Ganglioneuroma of adrenal gland in a patient with Turner syndrome. Ann Diagn Pathol.

[17] Pinsker JE, Crudo DF (2012). Ganglioneuroblastoma in a young child with Turner syndrome. J Pediatr Endocrinol Metab.

[18] Wikiera B, Nocoń-Bohusz J, Godziński J, Noczyńska A (2013). Ganglioneuroma in a patient with Turner syndrome. Pediatr Endocrinol Diabetes Metab.

[19] Jessop S, Lipsett J, Pal M, Connolly B (2022). A unique presentation of neuroblastoma in Turner syndrome: a case report. Pediatr Blood Cancer.

[20] Yang L, Yang Y, Qin Y, Feng YQ, Xie LL, Zhang DG (2022). Characteristics and karyotype analysis of a patient with turner syndrome complicated with multiple-site tumors: a case report. Open Life Sci.

[21] Schoemaker MJ, Swerdlow AJ, Higgins CD, Wright AF, Jacobs PA (2008). Cancer incidence in women with Turner syndrome in Great Britain: a national cohort study. Lancet Oncol.

[22] Boguszewski MC, Cardoso-Demartini AA, Boguszewski CL, Chemaitilly W, Higham CE, Johannsson G, Yuen KC (2021). Safety of growth hormone (GH) treatment in GH deficient children and adults treated for cancer and non-malignant intracranial tumors-a review of research and clinical practice. Pituitary.

[23] Bolar K, Hoffman AR, Maneatis T, Lippe B (2008). Long-term safety of recombinant human growth hormone in turner syndrome. J Clin Endocrinol Metab.

